# Estimating HIV Incidence among Adults in Kenya and Uganda: A Systematic Comparison of Multiple Methods

**DOI:** 10.1371/journal.pone.0017535

**Published:** 2011-03-07

**Authors:** Andrea A. Kim, Timothy Hallett, John Stover, Eleanor Gouws, Joshua Musinguzi, Patrick K. Mureithi, Rebecca Bunnell, John Hargrove, Jonathan Mermin, Reinhard K. Kaiser, Anne Barsigo, Peter D. Ghys

**Affiliations:** 1 HHS-Centers for Disease Control and Prevention, Atlanta, Georgia, United States of America; 2 Imperial College, London, England, United Kingdom; 3 Futures Institute, Glastonbury, Connecticut, United States of America; 4 Joint United Nations Programme on HIV/AIDS (UNAIDS), Geneva, Switzerland; 5 Uganda Ministry of Health, Kampala, Uganda; 6 National AIDS Control Council, Nairobi, Kenya; 7 HHS-Centers for Disease Control and Prevention, Nairobi, Kenya; 8 DST/NRF Centre of Excellence in Epidemiological Modeling and Analysis, SACEMA, Stellenbosch, South Africa; 9 National AIDS and STD Control Programme, Kenya Ministry of Health, Nairobi, Kenya; University of Oxford, Viet Nam

## Abstract

**Background:**

Several approaches have been used for measuring HIV incidence in large areas, yet each presents specific challenges in incidence estimation.

**Methodology/Principal Findings:**

We present a comparison of incidence estimates for Kenya and Uganda using multiple methods: 1) Epidemic Projections Package (EPP) and Spectrum models fitted to HIV prevalence from antenatal clinics (ANC) and national population-based surveys (NPS) in Kenya (2003, 2007) and Uganda (2004/2005); 2) a survey-derived model to infer age-specific incidence between two sequential NPS; 3) an assay-derived measurement in NPS using the BED IgG capture enzyme immunoassay, adjusted for misclassification using a locally derived false-recent rate (FRR) for the assay; (4) community cohorts in Uganda; (5) prevalence trends in young ANC attendees. EPP/Spectrum-derived and survey-derived modeled estimates were similar: 0.67 [uncertainty range: 0.60, 0.74] and 0.6 [confidence interval: (CI) 0.4, 0.9], respectively, for Uganda (2005) and 0.72 [uncertainty range: 0.70, 0.74] and 0.7 [CI 0.3, 1.1], respectively, for Kenya (2007). Using a local FRR, assay-derived incidence estimates were 0.3 [CI 0.0, 0.9] for Uganda (2004/2005) and 0.6 [CI 0, 1.3] for Kenya (2007). Incidence trends were similar for all methods for both Uganda and Kenya.

**Conclusions/Significance:**

Triangulation of methods is recommended to determine best-supported estimates of incidence to guide programs. Assay-derived incidence estimates are sensitive to the level of the assay's FRR, and uncertainty around high FRRs can significantly impact the validity of the estimate. Systematic evaluations of new and existing incidence assays are needed to the study the level, distribution, and determinants of the FRR to guide whether incidence assays can produce reliable estimates of national HIV incidence.

## Introduction

Measuring HIV incidence or the rate of new HIV infections in a population over time is of paramount importance for proper planning and evaluation of HIV prevention programs. Several methods have been proposed for measuring HIV incidence in large areas, yet each presents specific challenges [1], [2].

The original “gold standard” method for measuring population-level HIV incidence is a prospective cohort study that measures the occurrence of new infections in a well-defined HIV-negative population followed over time and tested at regular intervals for HIV infection. These studies, however, are rare, difficult and expensive to implement, and prone to biases that could reduce generalizability of results.

Most developing countries approximate adult HIV incidence using mathematical models that relate observed HIV prevalence to HIV incidence, which make assumptions on the average survival of HIV-infected individuals and the effect of antiretroviral (ARV) treatment on survival [3], [4]. In countries with generalized epidemics, the primary sources of HIV prevalence data for these models are routine unlinked and anonymous HIV sero-surveys among pregnant women attending antenatal clinics (ANC) and nationally representative population-based surveys (NPS) with HIV testing, including demographic health surveys (DHS) and AIDS indicator surveys (AIS) [5]. NPS have also been used to derive age-specific HIV incidence rates in the general population using HIV prevalence data from two sequential surveys in the country [6], [7], [8], [9]. This method has been broadly validated through comparison with cohort measures of incidence and has been applied to several settings where two such surveys exist.

HIV incidence assays are a laboratory-based approach for detecting recently acquired HIV infection in cross-sectional samples of HIV-positive specimens and designed to estimate population-level HIV incidence [10], [11], [12]. These assays are based on the principle that antibody response to HIV infection matures over time and that immunological biomarkers of HIV disease progression can be used to distinguish recent from non-recent HIV infection. The ideal assay has the property that all HIV-infected persons will eventually produce a non-recent test result. The mean time it takes to cross over the defined threshold value defines the assay's duration of recency (ω) [13]. Incidence rates are estimated by combining the number testing as recent on the assay, the mean duration of recency for the assay, and the number at risk for recent HIV infection in an incidence formula [10], [14]. To correct for individuals in a population who fail to progress out of the stage marked as ‘recent’ by the assay [13], [15], the application of statistical adjustments in the incidence formula is required [14], [16], [17], [18]. A critical component to these adjustments is the assay's false-recent rate (FRR), defined as the probability that a chronically infected individual (that is, an HIV-infected individual infected >12 months) will misclassify as recent on the incidence assay. Some HIV-infected individuals that are undergoing treatment with ARVs will also misclassify as recent on the assay as a result of enhanced viral suppression and corresponding decrease in antibody response [19], [20], [21]. Because of the significant impact that ARV use can have on incidence assay test results, all FRR and incidence surveys should have the ability to detect individuals that are currently taking ARVs to appropriately account for these individuals in the analysis. Additionally, auxiliary information on markers of advanced infection that could potentially impact the FRR to a large degree, including duration of HIV infection, CD4 cell counts, and age should also be collected to determine whether FRR varies significantly by these factors [6].

The most commonly used FRR value to date was derived for the BED IgG capture enzyme immunoassay (hereafter referred to as the BED assay) among a cohort of post-partum women followed from 1997-2001 in Zimbabwe. This study calculated a FRR of 5.2% [CI 4.4, 6.1] [16] among 2,749 women with long-term infections that were presumably not taking ARV treatment. The Zimbabwe FRR had been recommended as the default FRR value to use in settings that do not have a locally-relevant estimate of the FRR for the BED assay [18]; however, the universal applicability of the Zimbabwe FRR is unclear given that the FRR was estimated to be 1.7% in neighboring South-Africa [23].

In Uganda, impressive declines in HIV prevalence were documented from peak prevalence in the early 1990s at >10% to an estimated 6% in the early 2000s [24], [25], [26], [27]. More recently, there is evidence that HIV prevalence and incidence are no longer decreasing [28]. In Kenya, HIV prevalence has not changed significantly in recent years, with national surveys showing HIV prevalence at 6.7% in 2003 and 7.1% in 2007 [29], [30]. National adult prevalence was estimated at 7.5% [uncertainty range: 7.0, 7.9] in 2008, using UNAIDS Estimates and Projections Package (EPP) [31].

As countries gather multiple sources of incidence data, there is an opportunity to synthesize these data to determine the best supported level of incidence and incidence trends in populations. We compared several approaches for estimating incidence levels and trends among adults in the general populations of Uganda and Kenya. We used the availability of four different types of incidence methods to draw comparisons between the approaches.

## Methods

### Available HIV Surveillance Data

HIV prevalence data for urban and rural ANC clinics were available for Uganda from 1990–2007 and for Kenya from 1990–2005. NPS with HIV testing were conducted in Uganda in 2004/2005 (UAIS) and in Kenya in 2003 (KDHS) and 2007 (KAIS). To allow comparisons across results obtained with different methods, we restricted the analysis to adults aged 15–49 years.

### Mathematically Modeled Incidence in the Year of the Survey: Estimation and Projection Package (EPP)/Spectrum Models of Incidence in the General Population

The EPP and Spectrum software packages are used widely by countries in sub-Saharan Africa to produce national estimates and projections for HIV/AIDS, including indirect estimates of national adult HIV incidence. For this analysis, EPP was used to fit a simple 4-parameter epidemiological model to observed HIV surveillance data from ANC, calibrated to HIV prevalence data from NPS, using a maximum likelihood method separately for urban and rural areas. Bayesian melding was used to generate multiple curves reflecting the uncertainty in the prevalence data [3]. Adult HIV incidence for men and women combined was calculated from HIV prevalence over time using assumptions about the average survival of HIV-infected people and the effect of ARV treatment on survival. Urban and rural curves were combined into a set of national adult incidence curves over time. Using the Spectrum software these time trends of incidence and prevalence were then combined with country-specific demographic information [4]. Monte-Carlo simulations varying the above inputs were used to generate uncertainty ranges about the incidence estimates.

### Mathematically Modeled Incidence in the Year of the Survey: Survey-derived Models of Incidence from NPS

A mathematical method for estimating incidence from two sequential NPS with HIV testing was applied [7], [8], [9]. In Kenya where two NPS (2003 KDHS and 2007 KAIS) were available [29], [30], we assumed that individuals of age 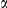 in the 2003 KDHS were represented by individuals aged 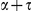 in the 2007 KAIS, where 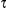 is the interval between surveys. The change in HIV prevalence among individuals of age 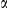 in the 2003 KDHS and in the 2007 KAIS was attributed to incident infections and AIDS deaths. The rate of AIDS death was based on the observed distribution of survival after HIV infection [32], and estimated HIV incidence for each age-group. In Uganda where only one NPS with HIV testing was available (2004/2005 UAIS), the same procedure was applied using a theoretical earlier survey to derive an earlier estimate of HIV incidence and assuming that HIV prevalence was constant in the five years preceding the survey. This assumption is consistent with data reported from national ANC surveillance [33] and a community-based cohort study in Uganda [28] which suggest stable HIV prevalence during that period. To the extent that real HIV prevalence rates were fluctuating, this estimate could be inaccurate and is considered in the interpretation of the results.

The effect of ARV use on HIV survival was accounted for by removing the fraction of HIV-infected individuals that were alive due to treatment in each survey [7]. To do this, data on ARV treatment scale-up in each country [34], [35], [36], [37] were used as were the assumptions that (1) ARV treatment was initiated one year before expected AIDS death [38] and (2) the age/sex-distribution of those on ARV was approximated by the distribution of AIDS deaths projected using Spectrum and published HIV prevalence trend data [4]. The survey-derived method does not account for the uncertainty introduced in the context of increased availability of ARV treatment. However, these assumptions have been validated previously [8], [39], [40]., Given that large scale up of ARV treatment programs have only recently occurred in Kenya and Uganda, we do not anticipate that ARV use significantly impacted the relevance of the mortality assumptions used in this analysis. A 95% bootstrap interval was calculated to quantify uncertainty in the incidence estimates due to random sampling errors in the HIV prevalence estimate.

### Assay-derived Incidence in NPS

The BED assay was applied to frozen HIV-positive dried blood spot samples from the 2003 KDHS [29] and serum samples from the 2007 KAIS [30] and the 2004/2005 UAIS [41]. Assay-derived HIV incidence and 95% confidence intervals (CI) were calculated using the recommended formula for assay-derived incidence estimation [15] and calibrated using the Zimbabwe FRR of 5.2% recommended for countries without local-derived FRR [16]. Estimates were also calibrated using a locally-derived FRR estimated from a cross-sectional sample of specimens from individuals residing in Rakai and Tororo districts in Uganda with chronic HIV infection and who were not known to be taking ARVs to treat their infection. Normally distributed errors around the FRR were assumed in the calculation of assay-derived incidence estimates.

A mean duration of recency of 155 days for the BED assay was applied to estimate annualized assay-derived incidence rates [42]. All assay-derived estimates in the NPS were weighted using individual sampling weights to generate national estimates of HIV incidence. Incidence estimates were adjusted to account for HIV positive specimens with missing BED assay test results, and 95% CIs around the estimates were calculated.

### Community Cohorts

A literature search of published papers and conference abstracts reporting HIV incidence rates from community-based cohort studies in Uganda and Kenya from 1990 to present was conducted. Three community-based cohort studies in rural Uganda, in Kayunga, Masaka and Rakai districts [28], [43], [44] were identified. Annual incidence rates (and their 95% CIs when reported) were abstracted by calendar year. For the Masaka cohort the average of published annual incidence rates for males and females aged >15 years was calculated and reported by calendar year.

### Trends in HIV Prevalence among Young Women Aged 15–24 Years Attending ANC Clinics, 2000–2007

HIV prevalence data collected from young pregnant women (aged 15–24 years) attending ANCs between 2000–2007 in Uganda and between 2000–2005 in Kenya were used as a proxy for HIV incidence trends in the general population [1]. Sites that were consistently included in national surveillance over time were included in the analysis. Regression analysis was used to assess the average change in prevalence per year in urban and rural areas. Linear regression provided the best fit to data in Uganda while exponential curves were fitted to the Kenyan data.

### Comparing Incidence Levels and Trends

Testing for differences in incidence level in a specific year was completed using the z-test statistic. Trends in EPP/Spectrum estimates from 2000–2005 in Kenya and 2000–2007 in Uganda were assessed for significance using a t-test statistic. Prevalence trends among young pregnant women attending ANC over time were considered statistically significant if the regression coefficients were significantly different from zero. HIV incidence estimates during the years of the NPS were compared to HIV prevalence estimates from the NPS to assess plausibility of the incidence level, using assumptions that national HIV incidence levels should not be substantially higher or lower than 10% that of national HIV prevalence levels in stable and mature epidemics.

## Results

### Calculation of a Local FRR in Samples of Known Long-term Infection

The Uganda FRR was estimated by pooling published data from FRR surveys in the Rakai Health Science Project (n = 473) from 2004–2007 and the Home Based AIDS Care program in Tororo District (n = 226) from 2003–2005 [20], [21]. Overall, among 699 specimens from HIV-infected persons who were known to be infected for >12 months and not known to be on ARV, 104 specimens classified as recent on the assay, resulting in a local FRR of 14.9% [CI 12.2, 17.5] for the BED assay.

### Comparison of Incidence Level

In the 2004/2005 UAIS, the total number of individuals participating in the NPS was 18,525. Of these, 1,092 were HIV-antibody positive, 1,023 had BED assay test results, and 172 tested recent on the assay. Availability of ARV treatment programs was presumed to be negligible in 2004 and not believed to have affected the BED assay test results. Weighted assay-derived incidence was 1.9% [CI 1.4, 2.3] using the Zimbabwe FRR and 0.3% [CI 0, 0.9] using the Uganda FRR (Table 1). The EPP/Spectrum incidence was 0.68% [uncertainty range: 0.61, 0.75] in 2004 and 0.67% [uncertainty range: 0.60, 0.74] in 2005. Survey-derived incidence was 0.6% [CI 0.4, 0.9] in 2005. Incidence in the Masaka cohort was 0.49% in 2004 and 0.25% in 2005 and prevalence was 7.7% [28]. In Rakai district, incidence was 1.24% for a prevalence of 11.4% in 2002, and in Kayunga district in 2007, incidence was 0.8% [CI 0.3, 1.2] for a prevalence of 9.9% [CI 8.6, 11.2] [43], [44]. National HIV prevalence from the 2004/2005 UAIS was 6.4% [CI 6.0, 6.7] [45].

**Table 1:** HIV Incidence and Prevalence Rates in the Year of the National Survey, by Estimation Method, Kenya and Uganda.

	Uganda				Kenya			
	2004		2005		2003		2007 f	
	Rate	95% range	Rate	95% range	Rate	95% range	Rate	95% range
**HIV Incidence** *								
EPP/Spectrum	0.68	0.61, 0.75	0.67	0.60, 0.74	1.04	1.03, 1.09	0.72	0.70, 0.74
Survey-derived†,‡			0.6	0.4, 0.9			0.7	0.3, 1.1
Assay-derived: Zimbabwe^a^			1.9	1.4, 2.3	2.5	1.7, 3.3	2.1	1.6, 2.6
Assay-derived: Ugandaa,b,c			0.3	0.0, 0.9	0.8	0.0, 1.8	0.6	0.0, 1.3
Cohort incidence^d^								
Masaka^e^	0.49		0.25					
**HIV Prevalence**			6.4	6.0, 6.7	6.7	5.8, 7.6	7.4	6.7, 8.1

*Among adults aged 15–49 years.

†Uganda 2000–2005.

‡Kenya 2003–2007.

a. All assay-derived estimates were weighted to account for unequal probability of selection and adjusted for non-response, where necessary. For the 2007 Kenya estimate, any participant that reported current ARV use was excluded from the incidence analysis.

b. The Uganda FRR was generated from: (1) pooled data from 699 ARV naive long-term specimens from Rakai (76/473) and rural Tororo districts (28/226) in Uganda that classified as false-recent on the BED assay.

c. Statistically significant difference observed in assay-derived estimate and the EPP/Spectrum estimate in Uganda 2005.

d. No data published on community cohort incidence in Kenya.

e. 95% confidence intervals not reported.

f. All participants that that reported current ARV use were excluded from the 2007 Kenya HIV prevalence estimate.

In the 2003 KDHS, the total number of survey participants was 5,994. Of these, 399 were HIV-antibody positive, 362 had BED assay test results, and 70 tested recent on the assay. Similar to Uganda, the availability of ARV treatment programs in Kenya in 2003 was insignificant to have made an impact on test results. Weighted assay-derived incidence was 2.5% [CI 1.7, 3.3] using the Zimbabwe FRR and 0.8% [CI 0, 1.8] using the Uganda FRR. The EPP/Spectrum incidence was 1.04% [uncertainty range: 1.03, 1.09]. National HIV prevalence from the 2003 KDHS was 6.7% [CI 5.8, 7.6] [29].

In the 2007 KAIS, the number of survey participants was 15,844. Of these, 1,098 were HIV- antibody positive, 876 had BED assay test results, and 151 were BED recent. A total of 92 participants that reported current ARV use and were later excluded from the incidence analysis. In 2007, EPP/Spectrum incidence was 0.72% [uncertainty range: 0.70, 0.74], survey-derived incidence was 0.7% [CI 0.3, 1.1], and weighted assay-derived incidence was 2.1% [CI 1.6, 2.6] and 0.6% [CI 0, 1.3] using the Zimbabwe and Uganda FRR, respectively. The KAIS reported a national HIV prevalence of 7.4% [CI 6.7, 8.1] in 2007 [30].

### Comparison of Trends

**Figure 1:**
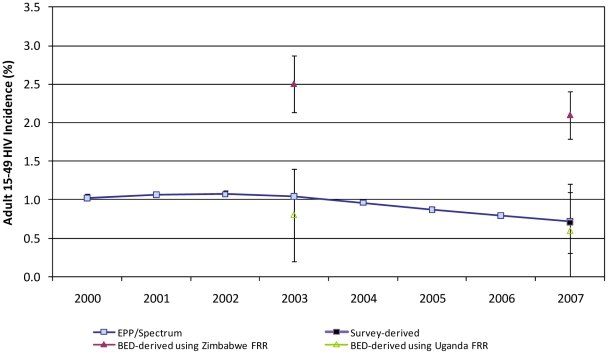
Trends in HIV incidence by estimation method, adults aged 15–49 years, Kenya, 2000–2007.

**Figure 2:**
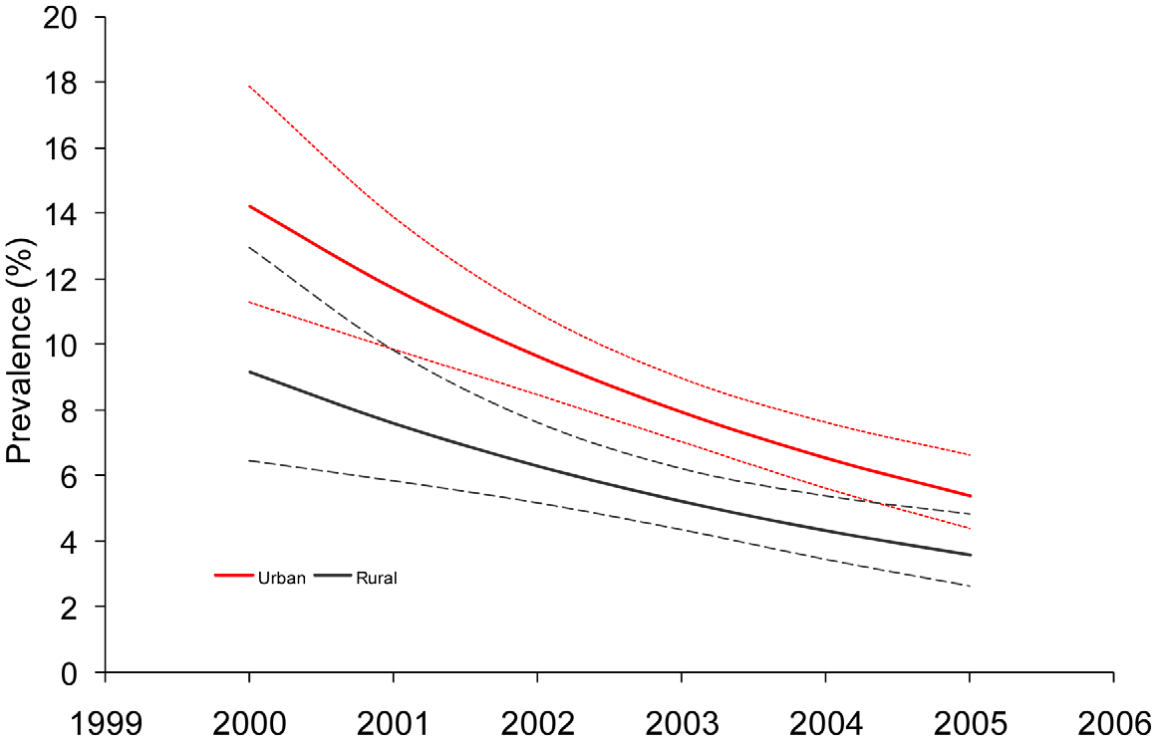
Trends in HIV prevalence among young women aged 15–24 years attending antenatal clinics in Kenya, 2000–2005.

In Kenya, the EPP/Spectrum incidence was stable at approximately 1% from 2000–2003 and declined significantly from 2003–2007, where incidence was estimated at 0.7% (Figure 1). Using the Zimbabwe FRR, assay-derived incidence was 2.5% in the 2003 KDHS and 2.1% in the 2007 KAIS. Using the Uganda FRR, assay-derived incidence was 0.8% in the 2003 KDHS and 0.6% in the 2007 KAIS. HIV prevalence among young ANC attendees aged 15–24 years in Kenya indicated a significant decline between 2000 and 2005 overall (p<0.001) and for both urban (p<0.001) and rural (p<0.001) areas (Figure 2).

**Figure 3:**
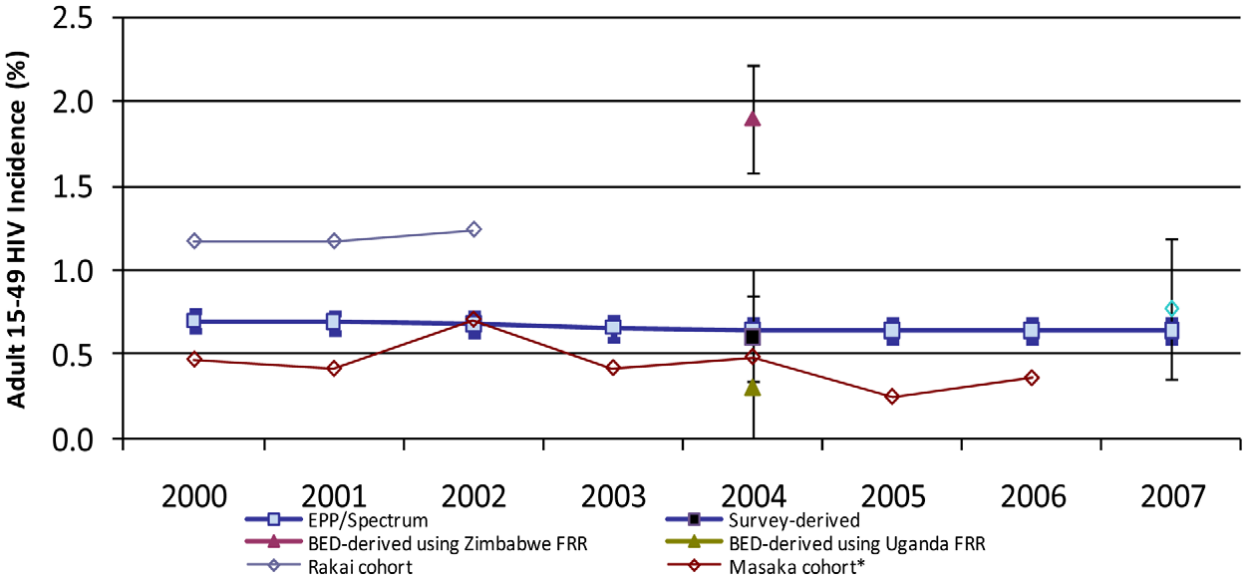
Trends in HIV incidence by estimation method, adults aged 15–49 years, Uganda, 2000–2007.

**Figure 4:**
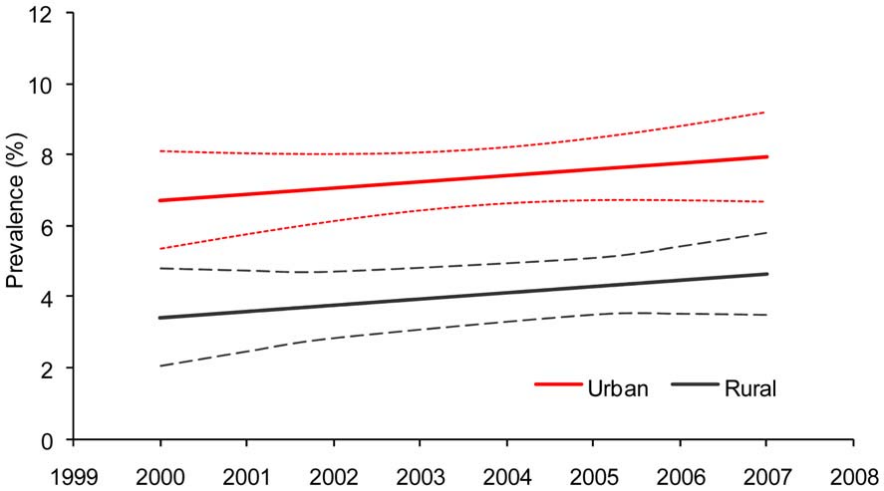
Trends in HIV prevalence among young women aged 15–24 years attending antenatal clinics in Uganda, 2000–2007.

In Uganda, EPP/Spectrum incidence remained stable at approximately 0.7% from 2000–2007 (Figure 3). In the Rakai cohort, HIV incidence was 1.2% in 2000 and 1.2% in 2002. In the Masaka cohort, HIV incidence was 0.5% in 2000, 0.7% in 2002, 0.5% in 2004, and 0.4% in 2006. HIV prevalence trends among young ANC attendees aged 15–24 years in Uganda showed a slight non-significant increase from 2000–2007 in both urban and rural areas (Figure 4).

## Discussion

Comparison of assay-derived incidence to modeled estimates of incidence provided evidence that when calibrating assay-derived incidence based on the Zimbabwe FRR of approximately 5%, assay-derived incidence estimates were inconsistent with those obtained by other methods in both Kenya and Uganda. The application of a local FRR of approximately 15% resulted in assay-derived incidence estimates that were reasonably consistent to estimates by other methods in Kenya. In Uganda, assay-derived estimates were two times lower than modeled estimates and similar to cohort-derived incidence reported in the same year. The differences observed were not statistically significant. In the analysis of incidence trends, results obtained by the different methods appeared to correspond fairly well with each other. In Uganda, incidence was stable from 2000–2007. In Kenya, incidence appeared to have declined since 2000 by all approaches.

Comparisons of prevalence and incidence levels in the three NPS confirm that the incidence estimates from all methods (i.e., EPP/Spectrum, the survey-derived method, and assay-derived method using the Uganda FRR) fell within plausible levels for Kenya in 2003 and 2007 (e.g., incidence estimates were 8–15% that of observed HIV prevalence in the same population). In contrast, in Uganda, the two mathematical models of incidence produced plausible levels of incidence (e.g., incidence estimates were approximately10% of prevalence), but assay-derived and cohort-derived incidence estimates were both lower, falling at approximately 4–5% of the prevalence level. The application of the Zimbabwe FRR produced implausible levels of incidence, at levels approximately 30–40% of prevalence, in all three surveys.

These findings confirm that BED assay-based incidence estimates must incorporate a FRR in the incidence calculation to account for false-recent classifications [14], [22], [46], [47]. There is less certainty, however, in choosing which FRR value to apply given the sensitivity of the incidence estimate to the value of the FRR. The Zimbabwe FRR had previously been shown to work well in the setting in which it was estimated in Zimbabwe [16], but did not result in reliable measures of incidence in Kenya and Uganda. Moreover, though the application of a local FRR from Uganda improved the plausibility of the assay-derived incidence estimate for both Kenya and Uganda, the wide confidence intervals around the estimates made it difficult to interpret these findings.

Given the widely differing values for BED FRRs obtained in studies with relatively large sample sizes in South Africa (1.7%), Zimbabwe, (5%), China (6%), and in Uganda (15%) [16], [21], [23], [48], it is clear that more BED FRR studies that are conducted systematically and powered sufficiently are needed to derive this factor in other settings before a determination can be made whether FRR values for the BED assay can be appropriately applied to estimate population-level incidence; whether a local FRR obtained in one country is applicable for all regions within a country and to other countries of close geographic proximity and similar HIV subtypes; and whether the value varies significantly over the course of the epidemic. Another parameter required for estimating assay-derived incidence is the assay's mean duration of recency. Evidence suggests that there may be significant variation in the mean duration of recency across various populations and HIV clades [16], [49]. If used consistently, the value of this parameter should not affect the analysis of incidence trends. However, because the incidence level will be impacted, local estimates of the mean duration of recency may be required to obtain accurate estimates of incidence in a given population. These issues highlight the need for systematic evaluations of the performance characteristics for new and existing incidence assays using standardized methods and well characterized specimen sets. Such an endeavor would require a central specimen repository to be established as a standard resource for these evaluations and to maximize comparability across assays. Specimens in this repository should cover large volumes of specimen panels from HIV seroconverter cohorts and individuals with chronic HIV infection across a wide of geographic settings, viral clades, and epidemic stages [50].

There is clear evidence that specimens from HIV-infected persons that are currently on ARV treatment have a high probability of falsely classifying as recent on an incidence assay and that this error varies significantly by time on ARV [20], [51]. Until an incidence assay that is not impacted by ARV use is available, current incidence assays should only be applied to settings where ARV use can be measured, either on the basis of survey participants' self-report [30] or by using laboratory methods to test for the presence of ARV markers in the blood. Though the latter approach may be more robust than self-report data, limitations still exist that can affect the accuracy of the test such as immediate metabolism of ARVs in the liver. Specimens that test recent on the assay but have confirmed evidence of ARV use can either be excluded from the incidence analysis or reclassified as non-recent on the assay to produce a valid estimate of incidence. While exclusion is an acceptable approach for analysis of assay-derived incidence data, it may result in uncertainty bounds that are wider than necessary [22]. Finally, care must be applied to ensure that the population targeted in the FRR survey and that for the incidence survey are similar with respect to demographics, HIV subtypes, epidemic history, and ARV treatment roll-out; else, the assay-derived incidence estimates will not be reliable. For example, if the FRR is estimated from specimens with persons with longstanding HIV infection and not on ARV, the incidence survey must also exclude such persons from incidence estimation.

The two indirect measurements of incidence in this analysis fell within a plausible range of HIV incidence in both countries. Though the survey-derived model was able to infer incidence using one NPS, this required an assumption of stable HIV prevalence in the preceding 5 years. This assumption was relevant for Uganda given documented evidence of stable HIV prevalence in the general population, but may not be for other countries considering this approach. If stable prevalence cannot be guaranteed for a given setting, it is recommended that this approach not be used until at least two NPS are available [7], [9].

The EPP/Spectrum estimate utilizes routinely collected data from ANC surveillance together with data from NPS to estimate national level adult incidence; therefore this approach remains an attractive method for estimating national incidence in generalized epidemics where these data are likely to exist. The advantage of mathematical models for incidence estimation is that they are easy to use, particularly if the model's input data can be easily accessed and are of good quality. A limitation, however, is that high quality data cannot be guaranteed for some countries due to incomplete reporting and lack of quality control measures in place. Additionally, a degree of uncertainty is associated with the modeled estimates given that they depend both on the structure of the model and on assumptions regarding key parameters which cannot always be determined directly from data for a specific country of interest. Though the assumptions in the EPP/Spectrum model are based on best available data, any errors in the model assumptions (example.g., with respect to survival of HIV-infected persons and ARV use) could impact the quality of the estimates. Further, at the time of writing these models have only been used to estimate incidence by age, sex and location but not by other characteristics (i.e., behaviors, marital status or income level) which may be useful for intervention planning. Finally, because both countries had collected nearly 20 years of ANC surveillance data and had completed one to two national HIV prevalence surveys, the corresponding prevalence and incidence estimates in the EPP/Spectrum models were constrained to narrow bounds which may not reflect the full uncertainty.

Prospective community cohort studies are commonly regarded as the “gold-standard” measure for community-level incidence because incidence can be directly observed in the sample. In this analysis, the main limitation of cohort studies is that they were conducted in limited geographical areas. The Rakai community cohort, for which only early years of incidence were available, reported substantially higher rates of incidence compared to other approaches for estimating population-level incidence. However the reported HIV prevalence level in Rakai in 2002 was nearly two times Uganda's national HIV prevalence in the 2004/2005 AIS. In contrast, the Masaka and Kayunga cohort studies, conducted in areas with lower prevalence than Rakai, reported incidence estimates that were lower than those observed in Rakai but consistent with the measures of incidence obtained with indirect methods for the same time period.

This analysis was subject to methodological issues that may have biased the interpretation of the results. First, the level of the Uganda FRR observed in this analysis was remarkably high. High levels of the FRR will result in large uncertainty in the assay-derived incidence estimate, rendering it difficult to interpret and use these data. Incidence assays that produce consistently low levels of the FRR in a variety of populations are optimal to ensure assays can reproduce valid estimates of incidence for all settings. To guide the development of improved incidence assays, a new target product profile has set the minimum acceptable value of a FRR at <2%, with a coefficient of variation <30%, for multiple HIV subtypes and geographic settings [22], [50]. Second, the Ugandan FRR was derived from adults residing in two geographic regions in Uganda (i.e., rural districts in Eastern and Southwestern Uganda) which may not have been representative of the broader national populations in this analysis and may have impacted the accuracy of the assay-derived estimates. To minimize this bias, the FRR should be estimated in a population that is representative of the one in which the incidence assay will be applied for incidence estimation. For example, if national incidence is desired, the FRR should be estimated in nationally representative samples. Additionally, the FRR may vary by the duration of the epidemic [6], [13], precluding the application of a standard local FRR over time. Though the Uganda FRR did not vary significantly by proxy variables for stage of HIV epidemic [e.g., duration of infection up to 12 years or by age (unpublished data)], given the uncertainty around the FRR, investigators should exhibit caution when applying this value and consider repeatedly measuring the FRR in a representative population over time. If this value is recent or there is evidence that the FRR does not vary over time, it can be incorporated into the incidence formula and expected to result in a significantly improved estimate. Moreover, if improved incidence assays can demonstrate consistently low FRR values in all settings, the need for continued measurement of the FRR prior to conducting incidence surveys will be greatly reduced [50]. Finally, this analysis did not report on age, sex, or geographic estimates of incidence, all of which are expected to vary substantially from national HIV incidence estimates in Kenya and Uganda.

The use of HIV prevalence among young pregnant women aged 15-24 years over time has been used as a surrogate measure for trends in incidence [1], [52]. As the onset of sexual activity in this age group is recent, prevalence is expected to reflect recent infections. However, a limitation in this approach is that it does not inform trends among men nor women aged >25 years. Depending on the surveillance system coverage, the data may not be representative of all regions of the country [53]. Nonetheless, we did find that observed trends in prevalence among ANC attendees aged 15–24 years corresponded well with observed trends in incidence in the overall population obtained through mathematical modeling and published cohort data.

In conclusion, in combination, multiple methods for estimating incidence in Kenya and Uganda appeared to converge in similar trend and levels, yet on an individual basis, each of the approaches have their limitations. It is evident that much work is still needed in the area of assay-derived incidence estimation. Systematic evaluations of incidence assays will help to determine whether this method can accurately and precisely measure incidence. Further, recent infection testing algorithms using a multiple incidence assays in combination with additional clinical (e.g., CD4 cell count, RNA testing), laboratory (e.g., ART testing), and historical information should be explored for improving the accuracy of assay-derived incidence estimates. Pending the development of improved incidence assays, we recommend triangulation of multiple methods for incidence estimation and interpretation of results in conjunction with other epidemiologic data (e.g., HIV prevalence in the same population) to assess plausibility of incidence trends and level in a country and use these data to improve programmatic and policy decisions in the national HIV response.
